# Exploring the Chemical Space of Macro- and Micro-Algae Using Comparative Metabolomics

**DOI:** 10.3390/microorganisms9020311

**Published:** 2021-02-03

**Authors:** Alison H. Hughes, Florent Magot, Ahmed F. Tawfike, Cecilia Rad-Menéndez, Naomi Thomas, Louise C. Young, Laura Stucchi, Daniele Carettoni, Michele S. Stanley, RuAngelie Edrada-Ebel, Katherine R. Duncan

**Affiliations:** 1 Strathclyde Institute of Pharmacy and Biomedical Sciences, University of Strathclyde, Glasgow G4 0RE, UK; a.hughes@strath.ac.uk (A.H.H.); magot.florent@gmail.com (F.M.); ahmed.tawfike@rothamsted.ac.uk (A.F.T.); Louise.c.young@strath.ac.uk (L.C.Y.); ruangelie.edrada-ebel@strath.ac.uk (R.E.-E.); 2 Department of Pharmacognosy, Faculty of Pharmacy, Helwan University, Cairo 11795, Egypt; 3 Scottish Association for Marine Science, Scottish Marine Institute, Oban PA37 1QA, UK; cecilia.radmenendez@sams.ac.uk (C.R.-M.); naomi.thomas@sams.ac.uk (N.T.); Michele.Stanley@sams.ac.uk (M.S.S.); 4 Culture Collection of Algae and Protozoa (CCAP), Scottish Marine Institute, Oban PA37 1QA, UK; 5 Axxam SpA, Openzone, Bresso, 20091 Milan, Italy; Laura.Stucchi.LS@axxam.com (L.S.); Daniele.Carettoni.DC@axxam.com (D.C.)

**Keywords:** microalgae, comparative metabolomics, biotechnology, bioactivity, mass spectrometry, microalgal metabolites

## Abstract

With more than 156,000 described species, eukaryotic algae (both macro- and micro-algae) are a rich source of biological diversity, however their chemical diversity remains largely unexplored. Specialised metabolites with promising biological activities have been widely reported for seaweeds, and more recently extracts from microalgae have exhibited activity in anticancer, antimicrobial, and antioxidant screens. However, we are still missing critical information on the distinction of chemical profiles between macro- and microalgae, as well as the chemical space these metabolites cover. This study has used an untargeted comparative metabolomics approach to explore the chemical diversity of seven seaweeds and 36 microalgal strains. A total of 1390 liquid chromatography-mass spectrometry (LC-MS) features were detected, representing small organic algal metabolites, with no overlap between the seaweeds and microalgae. An in-depth analysis of four *Dunaliella tertiolecta* strains shows that environmental factors may play a larger role than phylogeny when classifying their metabolomic profiles.

## 1. Introduction

Algae, both macro- and micro-, are extraordinarily rich in biological and chemical diversity, with more than 156,000 described species [1]. Photosynthetic eukaryotes diversified and underwent secondary (and even tertiary in the case of alveolates) endosymbiotic events. This led to a phylogenetically diverse group of organisms, with common ancestors, that span across four of the five clades on the eukaryotic tree of life [2]. Further adding to their biological diversity, algae are present across all latitudes, in a variety of habitats including freshwater and marine environments. For example, the European kelp forests of *Laminaria* spp. are dominated in the North by *L. digitata* whilst the Southern forest from Morocco to South England primarily consists of *L. ochroleuca* [3]. Although morphologically similar, there are differences in the abundance and diversity of their respective epibionts, with 57 of 130 associated species belonging exclusively to the *L. digitata* epibiont, which suggests that environmental variation has led to the divergence in these species.

Natural products, or specialised metabolites, are known to play a role in the adaptation of an organism to the specific complexities of their environment, and have been exploited by medicine since ancient times [4]. Metabolite extracts of seaweeds and eukaryotic unicellular microalgae have been found to have bioactive properties including antioxidant, anti-tumour, and antimicrobial [5,6,7]. For example, Rocha et al. screened 33 terpenes isolated from brown and red seaweeds and reported that one third of these exhibited a cytotoxic effect (IC_50_ <15 μM) against at least one cancer cell line [8]. Similarly, anticancer activity of microalgae has been shown. A study by Ingebrigsten *et al.* demonstrated that when cultured under a combination of low and high light irradiance and temperatures, the diatoms *Attheya longicornis, Chaetoceros socialis, Chaetoceros furcellatus, Skeletonema marinoi* and *Porosira glacialis* were active against melanoma cells A2058 [9]. Fractions from *A. longicornis* under all four culture conditions were active whilst only the high light-low temperature fractions of *C. socialis* had anticancer activity. In that same study, all species except *S. marinoi* showed activity in the protein tyrosine phosphatase 1B (PTP1B) assay, a negative regulator in the insulin signalling pathway that affects those with Type II Diabetes [9]. Yet it remains unknown how much overlap there is between natural products produced by seaweeds and those produced by their unicellular counterparts. In a recent United Nations (UN) report, 221 seaweed species were reported to have commercial value [10]. However, in the same analysis, only 10 microalgal species (not including cyanobacteria) were reported to have commercial value [11]. This gap in knowledge of the chemistry produced by microalgae is of particular interest in the blue biotechnology sector as microalgae have been reported to have applications in biofuels, cosmetics, nutraceuticals, and pharmaceuticals [12] At the same time, the demand for microalgal and cyanobacterial products as food supplements has already rapidly increased in recent years, with a global market of US$6.5 billion in 2017 [11].

Since many of the algal products on the market comprise crude or processed biomass, an untargeted approach is often used to analyse the chemical composition of these organisms [13]. Metabolomics, using liquid chromatography-mass spectrometry (LC-MS) or tandem mass spectrometry (LC-MS/MS) data, has been used for this purpose. This approach has been used to study the accumulation of starch in *Chlamydomonas reinhardtii* in response to changes in circadian rhythm [14], and the uptake of the metals selenium and iodine by cultures of *Chlorella sorokiniana* [15]. In order to gain an insight into the similarities and differences between crude metabolite extracts from the culture of different species, supervised statistical analyses, such as partial least squares discriminant analysis (PLS-DA), have been deployed [16]. This approach, coupled with approaches such as molecular networking, has been utilised in drug discovery, including the discovery of tutuilamides A–C, from a marine cyanobacterium, with potent elastase inhibition (IC_50_ 1–5 nM) and anticancer activity against lung H-460 cells [17]. Comparative metabolomics using the Global Natural Products Social (GNPS) molecular networking [18] platform led to the discovery of several new metabolites from cyanobacteria and microalgae, including yuvalamide A [19], pagoamide A [20], and palstimolide A, which exhibited strong anti-parasitic activity (IC_50_ of 223 nM against malaria and 4.67 μM against leishmaniasis) [21].

In the first part of this study, crude metabolite extracts of 20 microalgal strains, belonging to 15 genera and five phyla, were compared to crude metabolite extracts from seven seaweeds. Multivariate statistical analyses of mass spectral (MS^1^) data were used to compare the chemical profiles of these species and to investigate the variation in their profiles amongst different groups; (1) between seaweeds and microalgae, (2) between microalgae from different genera, and (3) between different species of the same genus (*Nannochloropsis, Chlamydomonas*, and *Diacronema*). In the second part of the study, 16 crude extracts from microalgae were studied to access the chemical differences between strains of the same species (for *Prymnesium parvum, Chrysotila carterae*, and *Dunaliella tertiolecta).* This analysis, coupled with metabolic indicator assays, showed the chemical diversity of seaweeds and microalgae, with a focus on their potential applications in drug discovery.

## 2. Materials and Methods

### 2.1. Strain Selection

A total of 43 specimens of macroalgae and microalgae, including seven macroalgal specimens, 14 Chlorophytes, 14 Haptophytes, three Ochrophytes, one Rhodophyte, and four diatomaceous strains isolated from marine and brackish environments were obtained from the Culture Collection of Algae and Protozoa (CCAP, Scottish Association for Marine Science, Oban, UK). Information on strain ID, environment, and isolation can be found in Table S1.

### 2.2. Culture Conditions and Metabolite Extraction

All cultures were maintained at 20 °C, 16:8 h light:dark cycle, 150 µmol/m^2^/s light intensity, and shaking at 100 rpm. The strains chosen for metabolomics analysis were pre-cultured (10% *v*/*v* inoculum in 100 mL, media specified in Table S1) and scaled-up in three 7-day increments until a 10% *v*/*v* inoculum in 400 mL medium was reached. This was further cultivated for 14 days with 4–5% *w*/*v* Dianion**^®^** HP-20 absorbent resin, previously activated with ethyl acetate, added to the cultures on day 12 and left for 2 days. On day 14, culture broth, cells and resin were harvested, centrifuged and the supernatant discarded. Cell and resin pellets were frozen (−80 °C) overnight and lyophilized (Scientific Laboratory Supplies, Yorkshire) until dry. Dry cell pellets were vortexed and subsequently extracted twice with ethyl acetate (100 mL) for 1 hr for each extraction. Extracts were dried in vacuo and the weights recorded. Strains were cultured in two batches (list of strains in each batch can be found in Table S1) following the same growth and extraction conditions specified above.

### 2.3. DNA Extraction and 18S rRNA Gene Amplification

An aliquot (2 mL) of liquid culture (microalgae) or macerated tissue (seaweeds) was centrifuged for 10 min (3822× *g*, SIGMA 1–14 microcentrifuge (Sigma-Aldrich Ltd., Dorset, UK)) to harvest sufficient cells for extraction. The supernatant was discarded and the cell pellets were frozen in liquid nitrogen and ground using a tissue lyzer. Genomic DNA was extracted using the DNeasy Plant Mini kit (QIAGEN, Hilden, Germany) according to the manufacturer’s instructions. DNA amplification and sequencing for ribosomal DNA was performed (see Table 1 for primers used) using the Taq PCR (polymerase chain reaction) Master Mix Kit (Qiagen, Hilden, Germany). The sequencing was performed on a 3730× lDNA Analyser and assembly of the sequence data was carried out using Geneious 6.1.7. Accession numbers for the all sequences are in Table S1.

### 2.4. Phylogenetic Analysis

Full and partial 18S rRNA gene sequences were sequenced in house or retrieved from the European Nucleotide Archive (Table S1) and aligned using multiple sequence comparison by Log-expectation (MUSCLE) [24]. Alignments were filtered to remove gaps caused by partial sequence data. A nearest neighbour-joining (NNJ) phylogenetic tree was constructed using the Tamura-Nei method [25] (1000 bootstraps) within Mega 7 (v.7.0.26) [26].

### 2.5. Bioassay Screening of Metabolite Extracts

*PPARα assay.* Human peroxisome proliferator-activated receptor alpha (PPARα; Gene ID: 5465) was cloned into the pcDNA-GAL4 vector (Stratagene, San Diego, CA, USA). The obtained construct was used for the transfection of the CHO GAL4-Luci cell line (Stratagene, San Diego, CA, USA) and the CHO-PPARα stable clone displaying the best pharmacological profile was obtained after three rounds of limiting dilutions. CHO-PPARα stable clone was seeded at 7500 cells/well in 384 MTP in Dulbecco’s modified Eagle’s medium (DMEM)/Nutrient MixF12 supplemented with sodium pyruvate, HEPES buffer, sodium bicarbonate, ultraglutamine-1 (BioWhittaker, Walkersville, MD, USA), foetal bovine serum (Euroclone, Milan, Italy), penicillin-streptomycin and G418-puromycin (InvivoGen, Toulouse, France). 24 h after seeding the medium was removed and Optimem (ThermoFisher Scientific, Waltham, MA, USA) was added, followed by test extracts and controls at 2X-concentration. Plates were incubated for 18 h at 37 °C, 5% CO_2_ and let equilibrate at room temperature for 1 h. The assay well volume was adjusted to 20 µL/well by using a CyBiWell dispensing unit (Analytic Jena, Jena, Germany) and a triton-luciferin mix was injected before reading the luminescent signal in kinetics for 120 sec with a FLIPR TETRA (Molecular Devices, San Jose, CA, USA). Data were initially analysed by Excel software (Microsoft, Redmond, WA) and Prism software (GraphPad, San Diego, CA, USA) while Screener^®^ software version 11.0.1 (Genedata AG, Basel, Switzerland) and Vortex software (Dotmatics, Bishop’s Stortford, UK) were used for comprehensive data analysis of screening data.

For the PPARα assay, area under the curve (AUC) of luminescence kinetics was normalized to percentage of activity by the following formula:

*% activity = (X−VC)/(SC−VC) × 100%*

where *X* is the AUC measurement of a certain well, *VC* is the median per plate of the vehicle control (buffer only) and SC is the median per plate of the stimulator control, represented by 10 μM WY14643 reference activator (EC_100_; Merck KGaA, Darmstadt, Germany). A value of 100% indicates complete activation of PPARα. For the selection of active extracts, a cut-off was computed as mean plus 3 standard deviations of the distribution of % activity of vehicle control wells.

*EL assay.* Human endothelial lipase gene (LIPG; Gene ID:9388; EL) was subcloned and expressed in insect cells with the baculovirus system. Briefly, full-length coding sequence of human EL was synthesized with codon-usage optimized for expression in insect cells (GeneArt Gene Synthesis; ThermoFisher Scientific; Waltham, MA, USA) and subcloned into pFastBac™ 1 expression vector into the SpeI/KpnI restriction sites in frame to a carboxyl-terminal poly-histidine tag using the Bac-to-Bac™ Vector System (ThermoFisher Scientific; Waltham, MA, USA). Recombinant bacmid DNA was obtained by transposition of pFastBac 1/EL into DH10Bac *E. coli* cells and used to transfect *S. frugiperda* Sf9 insect cells. High-titer baculovirus stock was obtained by two rounds of viral amplifications in Sf9 cells. Preparative recombinant expression of EL was performed at 3-litre scale (1 *×* 10^6^ cells/mL) at multiplicity of infection (MOI) 2 and time of infection (TOI) 48 h and the protein was recovered in SF-900 II SFM culture medium (ThermoFisher Scientific; Waltham, MA, USA). Samples of the cell culture media of the infected cells were resolved by sodium dodecyl sulfate polyacrylamide gel electrophoresis (SDS-PAGE) and analysed by western blot with anti-HIS antibodies (ThermoFisher Scientific, Cat #MA1-21315-HRP; Waltham, MA, USA) to confirm the presence of recombinant EL. Purification was performed from insect cell medium by IMAC affinity chromatography using the HisPur Ni-NTA Chromatography Cartridge (ThermoFisher Scientific, Waltham, MA, USA) and EL was eluted in 20 mM Tris-HCl pH 8.0, 100 mM Imidazole, 100 mM NaCl, 0.01% Triton X-100, 1 mM Pefabloc^®^ SC (Merck KGaA, Darmstadt, Germany). Elution fractions containing purified EL were assessed for catalytic activity (below) and stored in aliquots at −80 °C. The EL enzymatic reaction was assembled in 384-well microtiter plates (MicroPlate-384 non-binding, GreinerBio, 784,900; Merck KGaA, Darmstadt, Germany) in 50 mM Tris-HCl pH 8.0, 100 mM NaCl, 25 mM MgCl_2_, 0.05% bovine serum albumin (BSA) with 6 nM EL and 1.5 µM L-3000 substrate (Echelon Biosciences Inc.; Salt Lake City, UT) as follows: (1) addition of 10 µL 3X extracts/controls; (2) addition of 10 µL 3X Enzyme mix; (3) addition of 10 µL 3X Substrate mix. The reaction was incubated for 60 min at 30 °C and the fluorescent signal at λex 500 nm, λem 530 nm was measured in kinetics with the PHERAstar FSX (BMG Labtechnologies; Ortenberg, Germany). Data were initially analysed with Mars PHERAstar software (BMG Labtechnologies; Ortenberg, Germany), Excel (Microsoft, Redmond, WA), and Prism software (GraphPad, San Diego, CA, USA), while Screener^®^ software version 11.0.1 (Genedata AG, Basel, Switzerland) and Vortex software (Dotmatics, Bishop’s Stortford, UK) were used for comprehensive data analysis of screening data.

For the EL assay, relative fluorescent units (RFU) endpoint measurement at 60 min of reaction was normalized to percentage of activity by the following formula:

*% activity = (X−VC)/(IC−VC) × 100%*

where *X* is the endpoint measurement of a certain well, *VC* is the median per plate of the vehicle control (buffer only) and *IC* is the median per plate of the inhibitor control, represented by 1 μM Orlistat reference inhibitor (IC_100_; Merck KGaA, Darmstadt, Germany). A value of 100% indicates complete inhibition of EL. For the selection of active extracts, a cut-off was computed as mean plus 3 standard deviations of the distribution of % activity of Vehicle Control wells.

*PTP1B assay.* All materials were procured from Sigma Aldrich (Dorset, UK).

In a total volume of 40 µL, protein-tyrosine phosphatase 1B (PTP1B) (1 nM) was pre-incubated in the presence and absence of test compound or standard (bis(4-trifluoromethylsulfonamidophenyl)-1,4-diisopropylbenzine (Protein Tyrosine Phosphatase Inhibiter IV-TFMS)) at 37 °C for 30 min in 25 mM HEPES buffer containing sodium chloride 50 mM, dithiothreitol 2 mM, ethylenediaminetetraacetic acid (EDTA) 2.5 mM, BSA 0.01 mg/mL, catalase 250 µg/mL, pH7.2, in a half-area black 96-well plate. Subsequent to this, 6,8-difluoro-4-methylumbelliferyl phosphate (DiFMUP—substrate) (10 µM) in supplemented HEPES buffer, was added to the reactant mixture and incubated at 37 °C for a further 10 min. The resulting fluorescent signals were measured on a Wallac Victor 2 multilabel plate reader (Perkin Elmer, Beaconsfield, UK), in fluorescent mode: Excitation 360/Emission 460 nm. The enzyme substrate reaction in the absence of compound/extract was referred to as the control. The assay background was determined by measuring the fluorescence of substrate and buffer only. Bis(4-trifluoromethylsulfonamidophenyl)-1,4-diisopropylbenzine (Protein Tyrosine Phosphatase Inhibiter IV-TFMS) in the concentration range of 10 µM to 25 mM, was used as a standard compound to validate the assay system. The activity of the standard and test compounds was calculated by using the formula: % Inhibition = 100 − (Compound §RFU-Background RFU)/(Control RFU-Background RFU) × 100). § relative fluorescent units. Extracts were screened at a concentration of 30ug/mL. Data were analysed and expressed as a percentage of control (enzyme substrate reaction in the absence of any extract or compound). A threshold of activity (40% of the control, which is 60% inhibition) was designated as “active”.

### 2.6. Tandem High-Resolution Mass Spectrometry Data Acquisition

The two batches of extracts were run separately using the following protocol, however two different ACE C18 columns were used. Due to the change in column and the time lapsed (~2 years) between analysis of batch 1 and batch 2, these datasets have been analysed separately.

Crude metabolite extracts were dissolved in methanol to a final concentration of 1 mg/mL and injected onto an Accela HPLC (high-performance liquid chromatography apparatus, Thermo Scientific, Bremen, Germany) using ACE C18 reversed-phase HPLC column (75 × 3.0 mm, 5 μm; HiChrom, Reading, UK). Samples were analysed with a Finnigan LTQ Orbitrap spectrometer coupled to a Surveyor Plus LC system (Thermo Fisher, Bremen, Germany). A binary gradient of solvent A (Millipore water and 0.1% formic acid) and solvent B (acetonitrile and 0.1% formic acid) was utilised as follows: 0–30 min linear gradient 10–100% B, 30–36 min at 100% B, 36–45 min 10% B. The sample was injected (10 μL) with a flow rate of 300 μL/min, the tray temperature was maintained at 4 °C and the column oven at 20 °C. Data-dependent MS^2^ experiments were carried out in positive mode electrospray ionisation (ESI) using a 100–2000 *m*/*z* mass range and 30,000 resolution. Capillary voltage was 35 V, capillary temperature was 270 °C, ion spray voltage was 4.5 kV, and tube lens voltage was 110 V. Collision-induced dissociation (normalised collision energy 35%, activation Q 0.250 ms, activation time 30,000 ms) of the 1st, 2nd, and 3rd most intense peaks for MS^2^ was accomplished using an Orbitrap analyser with a resolution of 15,000 and minimum ion signal threshold of 500. Before use, the instrument was tuned (according to the manufacturer’s instructions) and calibrated using acetonitrile dimer and caffeine (positive ion mode). MS^2^ signals from batch 2 were not sufficiently amplified to allow informative MS^2^ analysis, therefore MS^1^ data was extracted for both batches and used for multivariate analysis. Raw LC-MS data files and filtered peaklists are publicly available at ftp://massive.ucsd.edu/MSV000086453/ (accessed on 13 November 2020).

### 2.7. Processing of Raw Liquid Chromatography-Mass Spectrometry (LC-MS) Data Using MZmine

Raw positive ionisation mode MS^1^ data were extracted and converted to mzML files using ProteoWizard MSconvert tool [27] and directly processed using MZmine 2.30 [28]. The noise level was set at 1000. Chromatogram building was achieved using a minimum time span of 0.5 min, minimum height of 10,000, and *m*/*z* tolerance of 0.01 (or 8 ppm). The local minimum search deconvolution algorithm was used with the following settings: chromatographic threshold = 90%, minimum retention time range = 0.4 min, minimum absolute height = 10,000, minimum ratio of peak top/edge = 2, and peak duration range = 0.2–5.0 min. Chromatograms were deisotoped using the isotopic peaks grouper algorithm with a *m*/*z* tolerance of 0.01 (or 8 ppm) and a RT (retention time) tolerance of 0.5 min. Peak alignment was achieved using an *m*/*z* tolerance of 0.01 (or 8 ppm), 5% relative retention time tolerance and a weight of 20 for *m*/*z* and retention time. The peak list was gap-filled with the peak finder module (intensity tolerance at 25%, *m*/*z* tolerance at 0.01 (or 8 ppm), and absolute RT tolerance of 0.5 min). Ions that appeared in solvent or media blanks were removed from the analysis and the resultant peak lists were exported as a csv file.

### 2.8. Multivariate Statistical Analysis

The peak intensity table was uploaded to MetaboAnalyst [29], and missing values were replaced with small values during the data integrity check. Data was log transformed before being normalised according to the median and auto-scaled. Supervised PLS-DA was performed and hierarchical clustering producedheatmaps of chemical profiles.

## 3. Results

**Figure 1:**
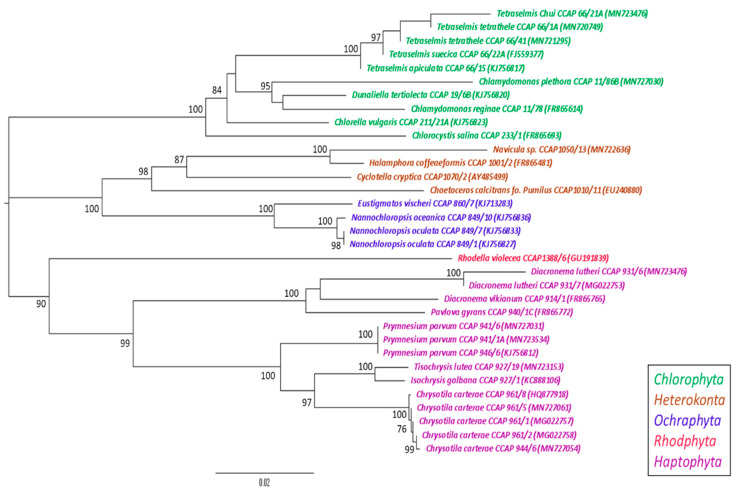
Nearest Neighbour-Joining phylogenetic tree of 18S rRNA microalgal genes. Taxa colouring represents the phyla; green, Chlorophyta; brown, Heterokonta; blue, Ochrophyta; red, Rhodophyta; purple, Haptophyta. Bootstrap values above 70% are indicated on the branches.

Microalgal strains belonging to the phyla Chlorophyta, Haptophyta, Ochrophyta, Rhodophyta, and Heterokonta (diatoms) were chosen for their diversity and the 18S rRNA gene sequence similarity of these strains were compared (Figure 1). In total, the 18S rRNA gene of 33 of the 36 microalgal strains selected for this study were sequenced and included in the phylogenetic analysis. In order to explore the diversity and chemical space occupied by microalgae, two separate comparative metabolomics analyses were performed. The first compared crude metabolite extracts from seaweeds and strains belonging to each phyla of microalgae, whilst the second analysis focused on species and strain diversity within the genera *Dunaliella, Chlamydomonas, Chrysotila, Prymnesium, Nannochloropsis* and *Diacronema*.

After establishing a phylogenetic relationship between microalgal strains based on 18S rRNA gene sequences, 20 strains of microalgae (seven Chlorophytes, four diatoms, six Haptophytes, two Ochrophytes, one Rhodophyte) and seven seaweed specimens were chosen for metabolomic comparison. Supervised statistical PLS-DA was used to compare the presence/absence and relative abundance of parent ions in each sample (Figure S1).

**Figure 2:**
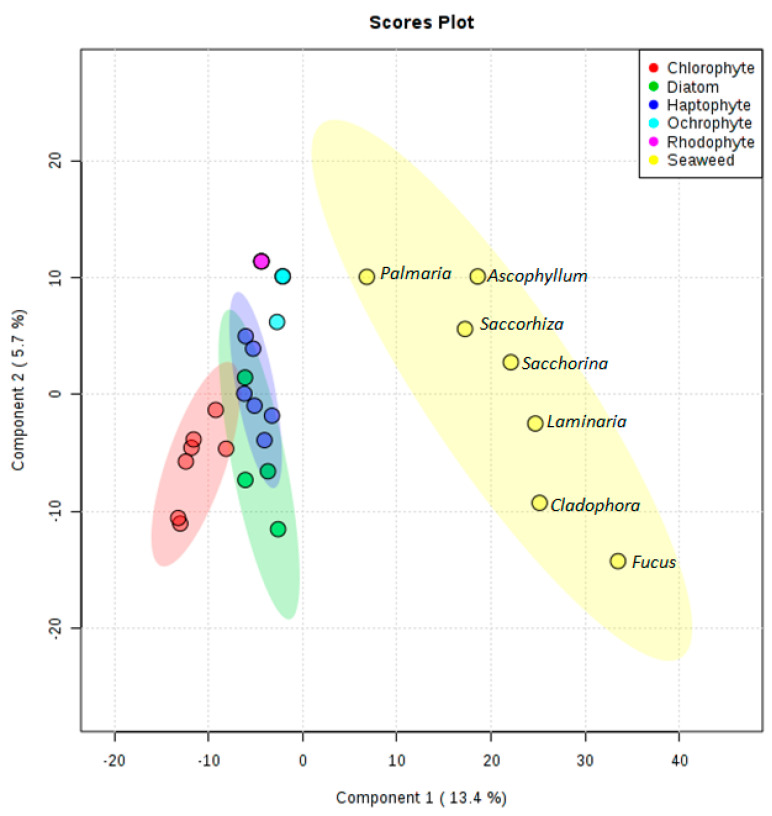
Partial least squares discriminant analysis (PLS-DA) scores plot for seaweed (yellow), Chlorophytes (red), diatoms (green), Haptophytes (dark blue), Ochrophytes (light blue), and Rhodophytes (pink) using component 1 (13.4%) and component 2 (5.7%) as axes.

Peaklists generated from filtered positive mode mass spectral data were analysed using MetaboAnalyst. Ions present in solvent and media blanks were removed from the analysis to prevent uninformative skewing of the results. We detected 1390 features between all 27 samples, with each feature representing a unique combination of the *m/z* value and chromatogram peak characteristics. It was observed in the metabolomics data that no metabolites were shared between the seaweed and the microalgal ethyl acetate extracts. On the other hand, depending on the phylum, the chemical diversity of the microalgae was similar (e.g., Diatom and Haptophyte) or appeared to expand into different spaces (e.g., Chlorophyte and Diatom) (Figure 2 and Figure S1). The number of features detected for seaweeds and Haptophytes were greater than the number of features detected across the other phyla (average of 435 and 370, respectively), with the Rhodophyte *Rhodella violacea* having the lowest number of detected features at 123. Metabolites extracted from the red seaweed *Palmaria palmata* occupy a similar chemical space to those extracted from the Rhodophyte *Rhodella violacea* illustrating that there is a relationship between the macro and micro algal forms within this phylum. Interestingly, the brown seaweeds *Ascophyllum nodosum, Saccorhiza polyschides* and *Saccharina latissima* cluster closely together in the PLS-DA plot despite belonging to different families (Fucaceae, Phyllariaceae, and Laminariaceae, respectively).

**Figure 3:**
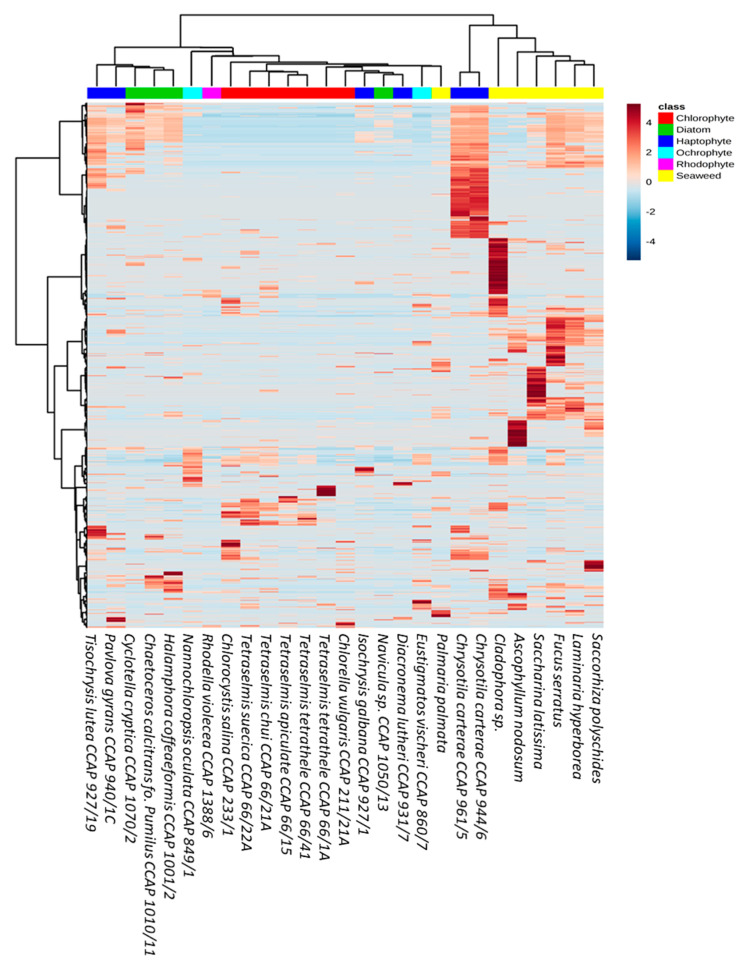
Hierarchical clustering of all detected ions across seaweeds (yellow), Chlorophytes (red), diatoms (green), Haptophytes (dark blue), Ochrophytes (light blue), and Rhodophytes (pink).Heatmap shows relative abundance (low; blue, high; red) of respective features.

It was observed that the chemical diversity expanded beyond taxonomic boundaries. Indeed, only the Chlorophyte metabolites clustered together whilst Haptophyte, Ochraphyte, and diatom samples did not cluster solely according to their phyla (Figure 3). The lower number of samples within the Ochrophyte and Rhodophyte clades meant that this pattern could not be confirmed. The Haptophyte clade was the most diverse, with *Chrysotila carterae* producing a greater abundance of low molecular weight metabolites (219–678 *m*/*z)* compared to the other Haptophyte strains screened (Figure 3). Although they clustered together, there were no distinct metabolite patterns observed to differentiate the five *Tetraselmis* strains from the other Chlorophytes, *Chlorocystis* and *Chlorella*.

**Figure 4:**
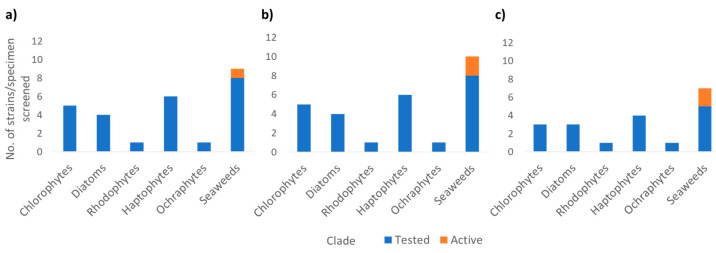
Bioassay screening results of extracts from each group tested (blue) and active (orange) for (**a**) endothelial lipase (EL), (**b**) proliferator-activated receptor alpha (PPARα), and (**c**) protein tyrosine phosphatase 1B (PTP1B). For the EL and PPARα assays extracts were selected as “active” if they fell within the mean plus 3 standard deviations of the distribution of % activity of vehicle control wells (Figure S3. For PTP1B assay A threshold of 60% inhibition was designated as “active”.

Crude metabolite extracts were screened against functional assays developed on three validated molecular drug targets: endothelial lipase (EL, LIPG), peroxisome proliferator-activated receptor alpha (PPARα) and protein tyrosine phosphatase 1B (PTP1B). In detail, endothelial lipase (EL; LIPG) plays a key role in atherosclerosis, and is actively investigated as a modulator in inflammatory processes and cancer [30,31], with examples of inhibitors of natural origin targeting closely related triacyglycerol lipases [32,33]. PPARα is a validated target for intervention in several therapeutic areas, including inflammation, diabetes, metabolic disorders and atherosclerosis [34,35], with specific agonists isolated from natural sources [36,37]. PTP1B acts as a negative regulator for the insulin signalling pathway and a drug target for the treatment of type II diabetes [38]. Due to low quantity of some microalgal metabolite extracts, not all strains were screened in each assay. Nevertheless, a total of 81 assays were performed (31 against EL, 31 against PPARα, and 19 against PTP1B). No activity was observed for the microalgal extracts but at least one seaweed extract was active in each bioassay (Figure 4). *Cladophora* sp. and *Fucus serratus* were active in the PPARα and PTP1B assay, whilst *Palmaria palmata* was the only extract active in the EL screen. These results are in agreement with those from the metabolomics analysis as *Cladophora* sp. and *Fucus serratus* occupy similar chemical space compared to *Palmaria palmata* which has a very different chemical profile.

**Figure 5:**
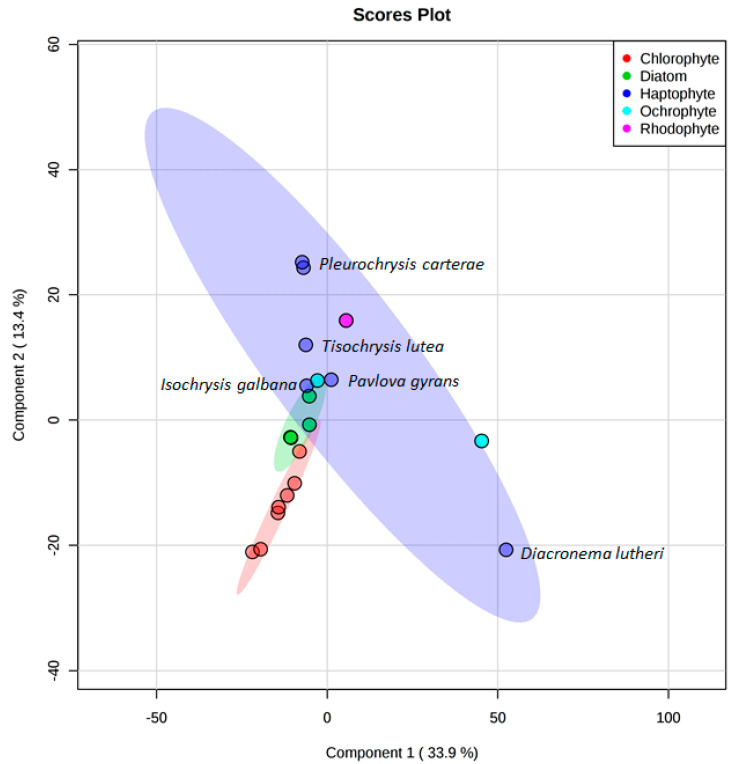
PLS-DA scores plot for Chlorophytes (red), diatoms (green), Haptophytes (dark blue), Ochraphytes (light blue), and Rhodophytes (pink) using component 1 (33.9%) and component 2 (13.4%) as axes.

The chemical diversity between microalgal phyla could be more clearly observed when the seaweed samples were removed from the PLS-DA (Figure 5). As already noted, the Haptophytes showed the greatest diversity with *Pleurochrysis carterae* and *Diacronema lutheri* occupying very different chemical spaces. Conversely, the strains belonging to Chlorophytes and diatoms cluster closely together. Whilst this can be explained for the Chlorophytes as five of the seven strains are *Tetraselmis* species, it is remarkable that the diatoms *Cyclotella cryptica, Chaetoceros calcitrans fo. pumilus, Halamphora coffeaeformis*, and *Navicula* sp. cluster so closely together. To the contrary, the strains *Isochrysis galbana, Pavlova gyrans, Cyclotella cryptica*, and *Eustigmatos vischeri*—from three different phyla—showed an overlap in their metabolite profiles.

**Figure 6:**
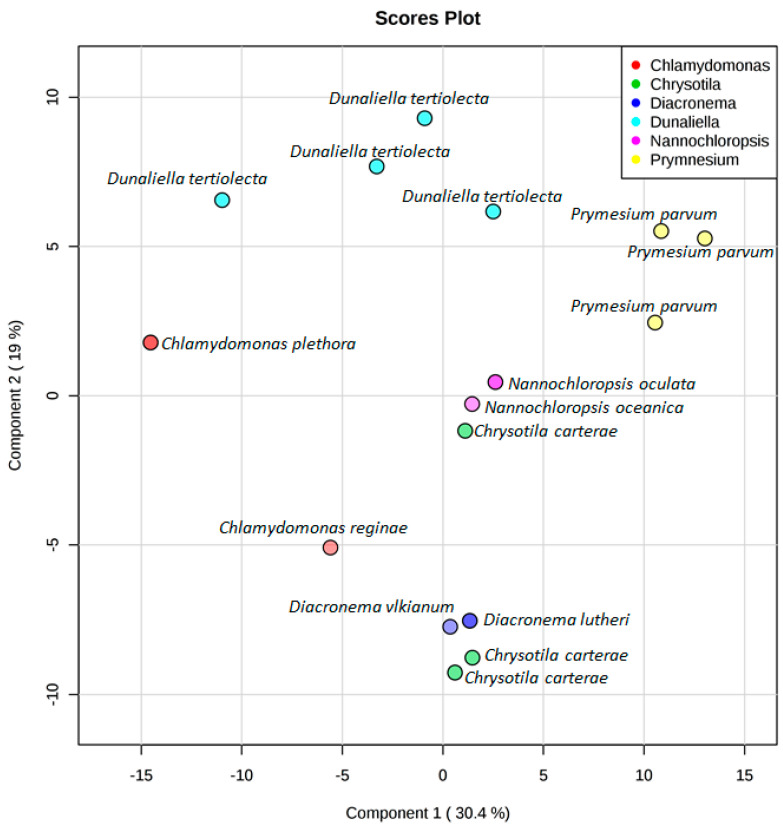
PLS-DA scores plot for *Chlamydomonas* spp. (red), *Chrysotila carterae* strains (green), *Diacronema* spp. (dark blue), *Dunaliella tertiolecta* strains (light blue), *Nannochloropsis* spp. (pink), and *Prymnesium parvum* strains (yellow) using component 1 (30.4%) and component 2 (19%) as axes.

A second comparative metabolomics experiment was designed to understand the diversity of microalgal chemical profiles on a species/strain level, often referred to as chemotypes. A total of 16 strains belonging to the genera *Dunaliella* (4), *Chrysotila* (3), *Chlamydomonas* (2), *Diacronema* (2), *Nannochloropsis* (2), and *Prymnesium* (3) were selected as Chlorophytes are a well-studied phylum and Haptophytes represented the greatest chemical diversity in the above analysis. Interestingly, the number of features detected in each of the samples varied considerably within species. The greatest variation was seen amongst the *Chrysotila carterae* strains with half the number of parent ions detected in CCAP 961/8 (46 features) compared to the CCAP 961/2 (83 features). Generally, a relationship between taxonomic classification and metabolite profiles could be observed; however, there were some anomalies (Figure 6 and Figure S2). *Chlamydomonas plethora* and *Chlamydomonas reginae*, as well as *Chrysotila carterae* CCAP 961/1 and *Chrysotila carterae* CCAP 961/8, belong respectively to the same genus and species, but do not share the same chemical space. In the case of the three *Chrysotila carterae* strains, CCAP 961/1 and 961/2 were isolated from marine environments whilst CCAP 961/8 was isolated from a brackish pool which may indicate that environment, rather than taxonomic classification alone, influences the metabolite profiles of these organisms. This trend was also observed for the four different strains of *Dunaliella tertiolecta*—strain CCAP 19/6B originated from a fjord in Norway, CCAP 19/7C came from the river Crouch in Essex, England, and the other two strains, CCAP 19/22 and CCAP 19/23, are from unknown marine locations. This supports arguments that geographical location and/or environment may have an influence on the chemotyping of strains belonging to the same species. In contrast to this, *Prymnesium parvum* strains cluster quite closely together despite two being from brackish waters (CCAP 941/1A and CCAP 941/6) and the third (CCAP 946/6) originating from a marine pool in Scotland.

**Figure 7:**
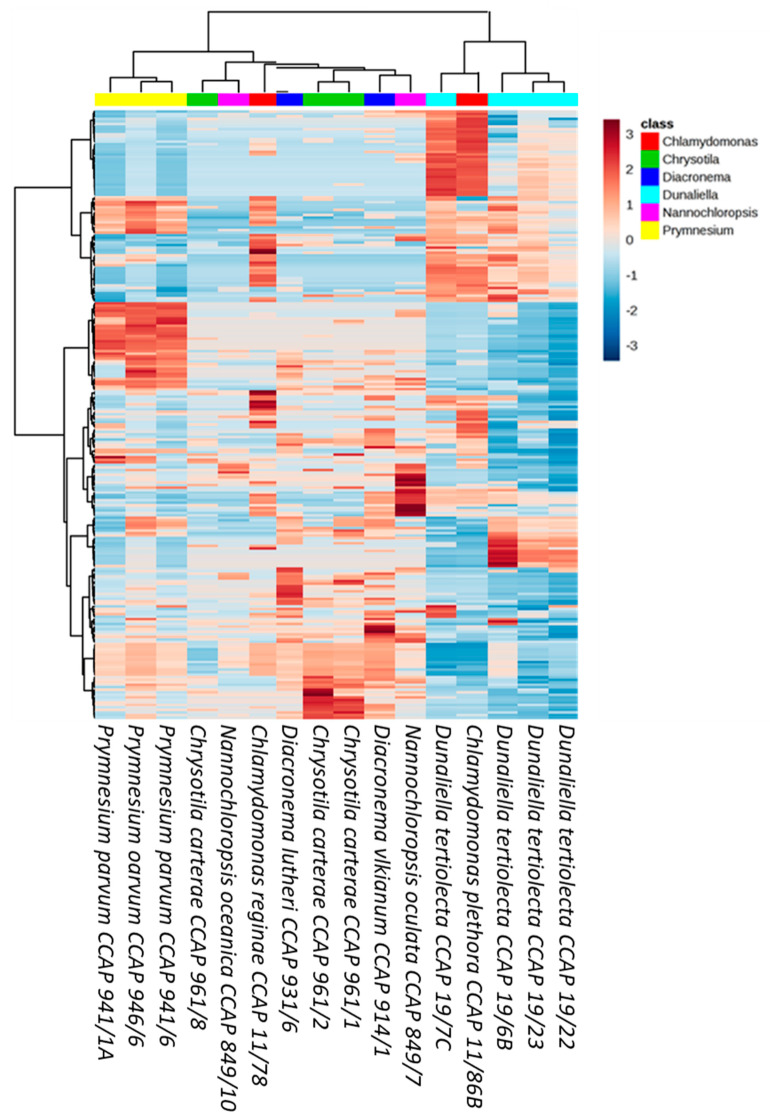
Hierarchical clustering of all detected ions across *Prymnesium* (yellow), *Chlamydomonas* (red), *Chrysotila* (green), *Diacronema* (dark blue), *Dunaliella* (light blue), and *Nannochloropsis* (pink). Heatmap shows relative abundance (low; blue, high; red) of respective features.

Unexpectedly, two species of *Nannochloropsis* (*N. oceanica*; hatchery, Norway, and *N. oculata*; lake of Tunis, Tunisia) clustered together in the PLS-DA scores plot despite the difference in the number of features detected (39 and 99, respectively). This difference is evident in the heatmap (Figure 7) with the following ions driving the variation between the two species; 455.2758 *m*/*z*, 317.2112 *m*/*z*, 183.6020 *m*/*z*, 367.2997 *m*/*z*, 383.3308 *m*/*z*, and 437.1515 *m*/*z.* The three marine strains of *Dunaliella tertiolecta* (CCAP 19/22, CCAP 19/23, and 19/6B) had similar chemical fingerprints, whilst CCAP 19/6B differed due to the absence of parent ions within the 202–370 *m/z* range (Figure 7).

## 4. Discussion

Based on the comparative metabolomics results, microalgae are a rich source of metabolites, many of which remain uncharacterised. Due to the applications of microalgal products in the biofuel and nutrition industries, there has been a large focus on investigating lipids and carotenoids produced by these organisms [39,40]. By using an untargeted metabolomics approach, we were able to illustrate that microalgae produce diverse suites of metabolites and that there is little evidence that a core metabolome exists that is shared across taxonomic boundaries. This is surprising as various classes of microalgae are distinguished by their carotenoid profiles, as well as morphology and genetic phylogeny [41]. This is also very exciting as macroalgae have been reported to have biological activities [42,43] that appear, from this study, to be distinct from those produced by their microalgal relations. However, since ethyl acetate was the solvent of choice for this analysis, many carotenoids were not efficiently extracted due to their polarity [44]. It is also interesting that from the 1390 ions analysed in this study, none were shared between seaweeds and microalgae which illustrates the biotechnological potential of microalgae as a source of chemistry separate to their macroalgal counterparts. This study gives a snapshot of the metabolites produced under one set of culturing conditions and extracted using a single solvent, and there is still much to be explored to gain true insights into the metabolomes of these organisms. A study by Luzzatto-Knaan et al. obtained over 15 million ultra-high performance liquid chromatography–tandem mass spectrometry (UPLC-MS/MS) spectra from 2600 fractions belonging to cyanobacteria, and algae compared to Actinobacteria (marine and terrestrial) and lichens reported that 86.3% of chemical features were unique to cyanobacteria and algae., and from this only 0.04% of those metabolites could be identified through the GNPS libraries which hold mass spectral data on more than 18,000 compounds [19]. Untargeted metabolomics and comparative techniques are powerful tools in gaining insights into the potential chemical space and biotechnological applications of microalgae.

With almost 160,000 extant species of algae, it is expected that their vast biological diversity will translate into chemical diversity. Haptophytes, in particular, were rich in chemistry with over 300 features detected from each strain, and represent an under-studied phylum in terms of biotechnological potential. The majority of species described within the phylum are coccolithophores, which are abundant in the marine environment as they form chalk deposits. Other species belonging to this phylum that are commonly studied are *Prymnesium* and *Phaocystis*, which form toxic algal blooms and use allelopathic strategies to achieve this [45]. Metabolomics approaches to investigating their chemical profiles could also be used to predict favourable conditions for algal bloom formations or aid in the identification of stresses that trigger the production of algal toxins. Due to Haptophytes’ involvement in chemical warfare, it is not surprising that they produce a plethora of metabolites with specialised functions that could be utilised in biotechnology and pharmaceutical sectors. Despite the lack of bioactivity observed for microalgal extracts in this study, there is a excess of literature reporting bioactive extracts and fractions from microalgae. However, from the entirety of the SeaBioTech programme, 927 microbial extracts were screened with only 36 testing positive in the PPARα assay and 118 testing positive in the EL assay [46]. By investigating the role of stress in eliciting the production of toxins and other specialised metabolites, the bioactivity profiles of these organisms may be unlocked through techniques such as One Strain MAny Compounds (OSMAC) [47] which can be complemented using comparative metabolomics.

This study also demonstrates the importance of environmental conditions when studying secondary metabolites. The disparity in chemical profiles among multiple strains of *Dunaliella tertiolecta* suggests that chemotyping organisms may be more important than phylogenetic identification when exploring chemical diversity. The existence of chemotypes means that care must be taken to report strain reference information or exact isolation details in publications pertaining to microalgal chemistry. Comparative metabolomics, particularly tools such as GNPS, MS2LDA [48], and feature-based molecular networking [49], have revolutionised how we visualise, interpret, and prioritise metabolites for applications within the biotechnology and pharmaceutical industries. Bioactivity-linked molecular networking led to the discovery of two potent chikungunya viral replication inhibitors (EC50 = 0.40 μM and 0.6 μM) isolated from *Euphorbia dendroides* that had been overlooked in previous analysis of the plant due to low abundance [50].

## 5. Conclusions

Seaweeds, microalgae, and often cyanobacteria are covered under the blanket term of “algae”which can lead to their biological and chemical diversity being overlooked. Comparative metabolomics is a useful tool in understanding and exploring the chemical space of microalgae, as well as their macroalgal and bacterial relations. From this study, it can be seen that there is a great disparity in metabolites produced by microalgae and seaweeds. It has also highlighted the potential geographical and/or environmental diversity of these organisms and that this, as well as taxonomy, influence the specialised metabolite profiles of microalgal strains. This opens the potential to study the effect of biotic and abiotic stress as a way to elicit the production of specialised metabolites and comparative analyses can guide the prioritisation and characterisation of bioactive metabolites as drug leads.

## Figures and Tables

**Table 1:** Primers used for 18S rRNA gene amplification and sequencing.

Name	Sequence
PCR	-
EAF3	TCGACAATCTGGTTGATCCTGCCAG [22]
ITS055R	CTCCTTGGTCCGTGTTTCAAGACGGG [22]
Sequencing	-
E528F	TGCCAGCAGCYGCGGTAATTCCAGC [22]
920F	GAAACTTAAAKGAATTG [22]
920R	ATTCCTTTRAGTTTC [22]
BR	TTGATCCTTCTGCAGGTTCACCTAC [22]
536R	GWATTACCGCGGCKGCTG [22]
GF	GGGATCCGTTTCCGTAGGTGAACCTGC [23]
GR	GGGATCCATATGCTTAAGTTCAGCGGGT [23]

## Data Availability

18S rRNA gene sequences are available at the accession number stated in Table S1. Raw mass spectral files are publicly available at ftp://massive.ucsd.edu/MSV000086453/.

## Supplementary Materials

The following are available online at https://www.mdpi.com/2076-2607/9/2/311/s1, Table S1: Strain information including 18S rRNA gene sequence accession numbers, Figure S1: Bar chart specifying number of MS features detected for each sample in Figure 2 from main text, Figure S2: Bar chart specifying number of mass spectrometry (MS) features detected for each sample in Figure 5 from main text. Figure S3: Example of primary screening on extracts in PPARα assay.
