# Supplementary material for: Exploring the Chemical Space of Macro- and Micro-Algae Using Comparative Metabolomics
Source: Microorganisms. 2021 Feb 3;9(2):311. doi: 10.3390/microorganisms9020311 (PMC7913273; doi:10.3390/microorganisms9020311)
Supplement: Supplementary file 1 [file microorganisms-09-00311-s001.pdf]

Supplemental Information

Table 1 – Details of the strains used in this study including taxonomy, 18S rRNA gene sequence accession numbers, origin of isolate and the media in which it was grown.

CCAP Code	Species	Phylum	Accession Number	Media	Origin
**Batch 1**					
66/15	*Tetraselmis apiculata*	Chlorophyta	KJ756817	f/2	Brackish; salt marsh, Marine House, Lincolnshire, England, UK
66/22A	*Tetraselmis suecica*	Chlorophyta	FJ559377	f/2	Brackish; River Alde, Suffolk, England, UK
66/1A	*Tetraselmis tetrathele*	Chlorophyta	MN720749	f/2	Brackish; salt marsh, Brancaster, Norfolk, England, UK
66/41	*Tetraselmis tetrathele*	Chlorophyta	MN721295	f/2	Marine; Vancouver Island, British Columbia, Canada
1010/11	*Chaetoceros calcitrans fo. pumilus*	Diatom	EU240880	f/2	Marine; Urayasu, Chiba Prefecture, Japan
1001/2	*Halamphora coffeaeformis*	Diatom	FR865481	f/2 + Si	Brackish; tidal pool, La Jolla, California, USA

233/1	*Chlorocystis salina*	Chlorophyta	FR865693	f/2	Marine; Ulva culture from Soulac, France
860/7	*Eustigmatos vischeri*	Ochrophyta	KJ713283	3N-BBM+V	Soil; Brixen, Austria
211/21A	*Chlorella vulgaris*	Chlorophyta	KJ756823	f/2	Brackish; River Crouch, Althorne, Essex, England, UK
940/1C	*Pavlova gyrans*	Haptophyta	FR865772	f/2	Marine; Cardigan Bay, Wales, UK
944/6	*Chrysotila carterae*	Haptophyta	MN727054	f/2	Marine; Station L2, English Channel
961/5	*Chrysotila carterae*	Haptophyta	MN727061	f/2	Marine; Port Erin, Isle of Man, British Isles
927/19	*Tisochrysis lutea*	Haptophyta	MN723153	f/2	Marine; Tahiti, Society Islands,
1050/13	*Navicula sp.*	Diatom	MN722636	f/2 + Si	Marine; Porcupine Abyssal Plain, North Atlantic
849/1	*Nannochloropsis oculata*	Ochrophyta	KJ756827	f/2	Marine; Skate Point, Isle of Cumbrae, Scotland, UK
1070/2	*Cyclotella cryptica*	Diatom	AY485499	f/2 + Si	Brackish; West Tisbury, Great Pond, Martha's

					Vineyard, Massachusetts, USA
66/21A	*Tetraselmis chui*	Chlorophyta	MN723167	f/2	No record
931/7	*Diacronema lutheri*	Haptophyta	MG022753	f/2	Marine; pools, nr. pier, Millport, Isle of Cumbrae, Scotland, UK
927/1	*Isochrysis galbana*	Haptophyta	KC888106	f/2	Marine; fish pond, Port Erin Marine Station, Isle of Man, British Isles
1388/6	*Rhodella violacea*	Rhodophyta	NA	f/2	Slightly brackish; öland Island, Baltic Sea, Sweden
no SAMS ID	*Cladophora*	Seaweed	NA	N/A	No record
no SAMS ID	*Ascophyllum Nodosum*	Seaweed	NA	N/A	No record
no SAMS ID	*Saccorhiza polyschides*	Seaweed	NA	N/A	No record
no SAMS ID	*Saccharina latissima*	Seaweed	NA	N/A	No record
no SAMS ID	*Palmaria palmata*	Seaweed	NA	N/A	No record

no SAMS ID	*Laminaria hyperborea*	Seaweed	NA	N/A	No record
no SAMS ID	*Fucus serratus*	Seaweed	NA	N/A	No record
**Batch 2**					
931/6	*Diacronema lutheri*	Haptophyta	MN723476	f/2	Marine; pools, nr. pier, Millport, Isle of Cumbrae, Scotland, UK
914/1	*Diacronema vlkianum*	Haptophyta	FR865765	f/2	Marine; sea water, Ryde, Isle of Wight, England, UK
961/1	*Chrysotila carterae*	Haptophyta	MG022757	f/2	Marine; off Plymouth, Devon, England, UK
961/8	*Chrysotila carterae*	Haptophyta	HQ877918	f/2	Brackish pool, Dunstaffnage Castle, Oban, UK
961/2	*Chrysotila carterae*	Haptophyta	MG022758	f/2	Marine; Station L2, English Channel
941/1A	*Prymnesium parvum*	Haptophyta	MN723534	f/2	Brackish; River Stour, Manningtree, Essex, England, UK

946/6	*Prymnesium parvum*	Haptophyta	KJ756812	f/2	Marine; pool, nr. Pier, Millport, Isle of Cumbrae, Scotland, UK
941/6	*Prymnesium parvum*	Haptophyta	MN727031	f/2	Brackish; River Stour, Manningtree, Essex, England, UK
19/6B	*Dunaliella tertiolecta*	Chlorophyta	KJ756820	f/2	Brackish; Oslo Fjord, Norway
849/10	*Nannochloropsis oceanica*	Ochrophyta	KJ756836	f/2	Marine;
19/7C	*Dunaliella tertiolecta*	Chlorophyta	KJ094615<br><br>(partial sequence, not included in phylogenetic analysis)	f/2	Brackish; River Crouch, Essex, England, UK
19/22	*Dunaliella tertiolecta*	Chlorophyta	KJ094626<br><br>(partial sequence, not included in phylogenetic analysis)	2ASW	Marine
19/23	*Dunaliella tertiolecta*	Chlorophyta	KJ094627<br><br>(partial sequence, not included in phylogenetic analysis)	2ASW	Marine
11/78	*Chlamydomonas reginae*	Chlorophyta	FR865614	f/2	Marine; Per Haridy, Roscoff, France

11/86B	*Chlamydomonas plethora*	Chlorophyta	MN727030	SNA	Brackish; Butley River, Aldeburgh, Suffolk, England, UK
849/7	*Nannochloropsis oculata*	Chlorophyta	KJ756833.1	SNA	Marine; Lake of Tunis, Tunisia

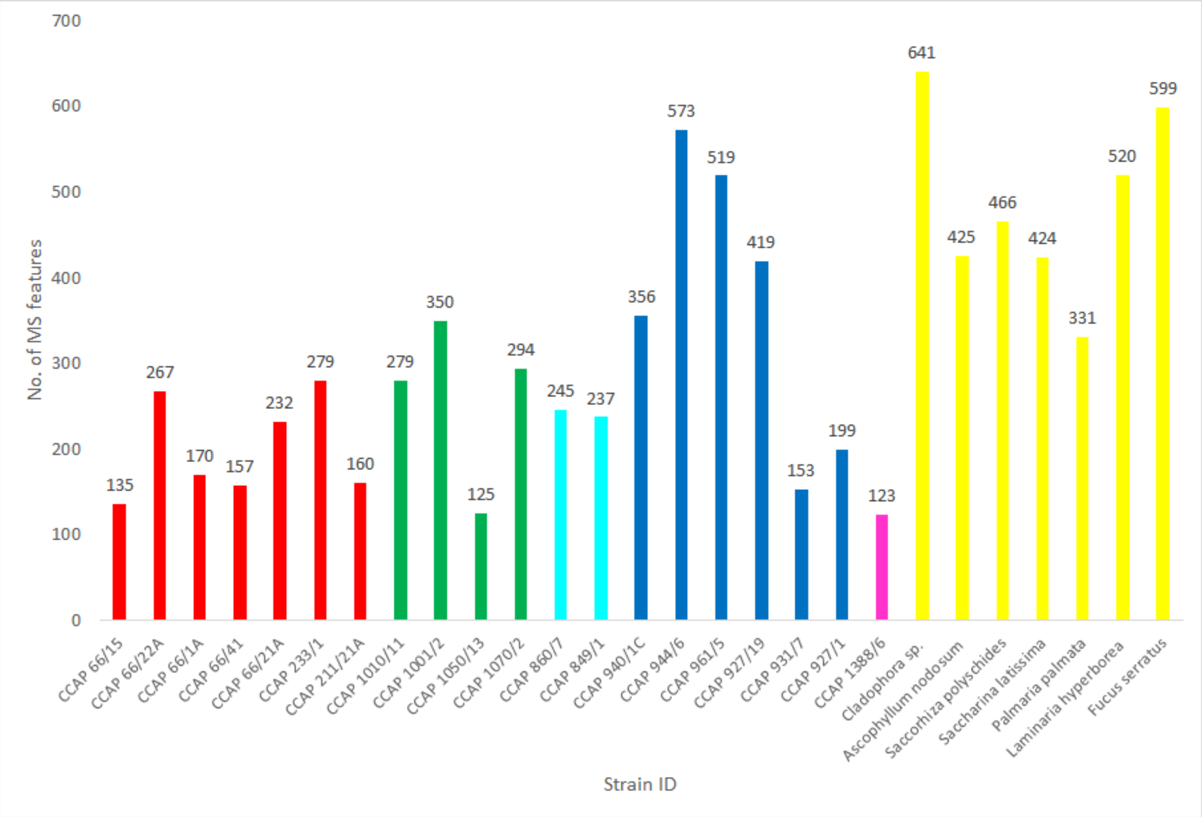

SI Figure 1 - Bar chart of the number of MS features detected in MetaboAnalyst for each strain/specimen used in Figure 2. Strain or specimen ID is given on the x-axis and the number of features detected from each extract is indicated on the bar chart. Grouping is according to taxonomy; seaweeds (yellow) and microalgal phyla; Chlorophytes (red), diatoms (green), Haptophytes (blue), Ochrophytes (light blue), and Rhodophytes (pink).

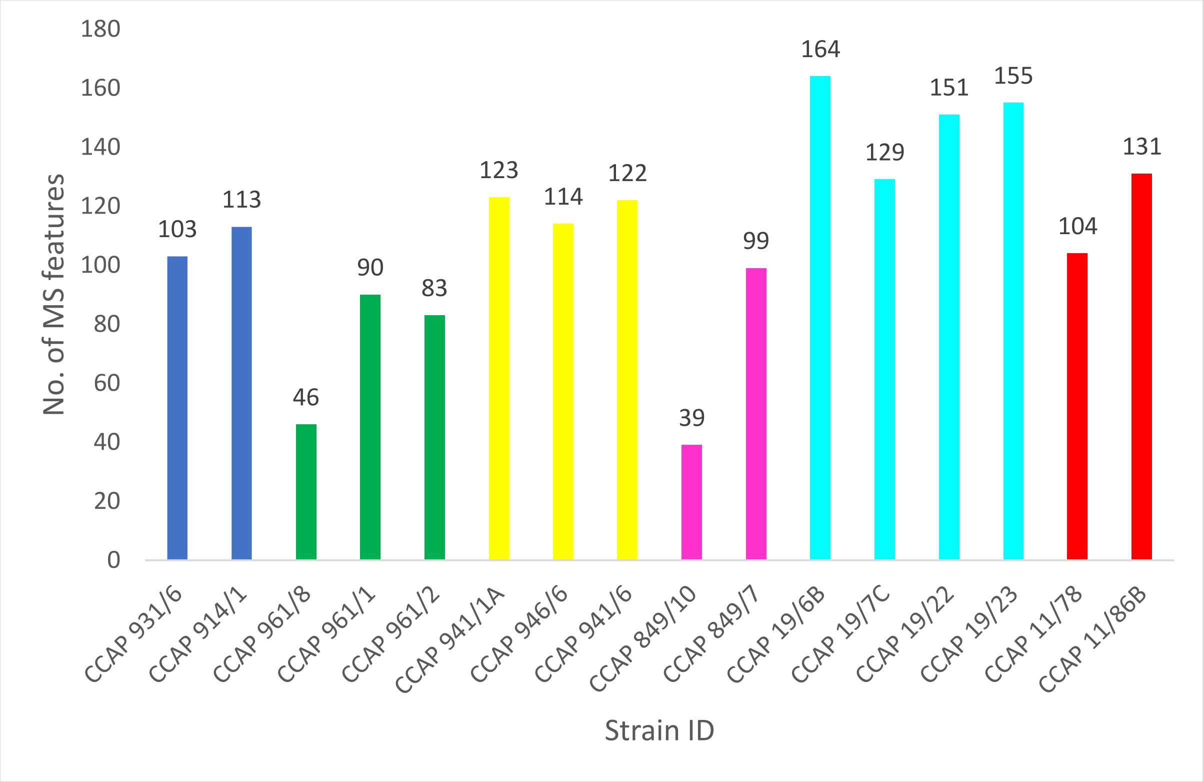

SI Figure 2 - Bar chart of the number of MS features detected in MetaboAnalyst for each strain/specimen used in Figure 6. Strain or specimen ID is given on the x-axis and the number of features detected from each extract is indicated on the bar chart. Grouping is according to taxonomy; *Diacronema* (blue), Chrysotila (green), *Prymnesium* (yellow), *Nannochloropsis* (pink), Dunaliella (light blue), and *Chlamydomonas* (red).

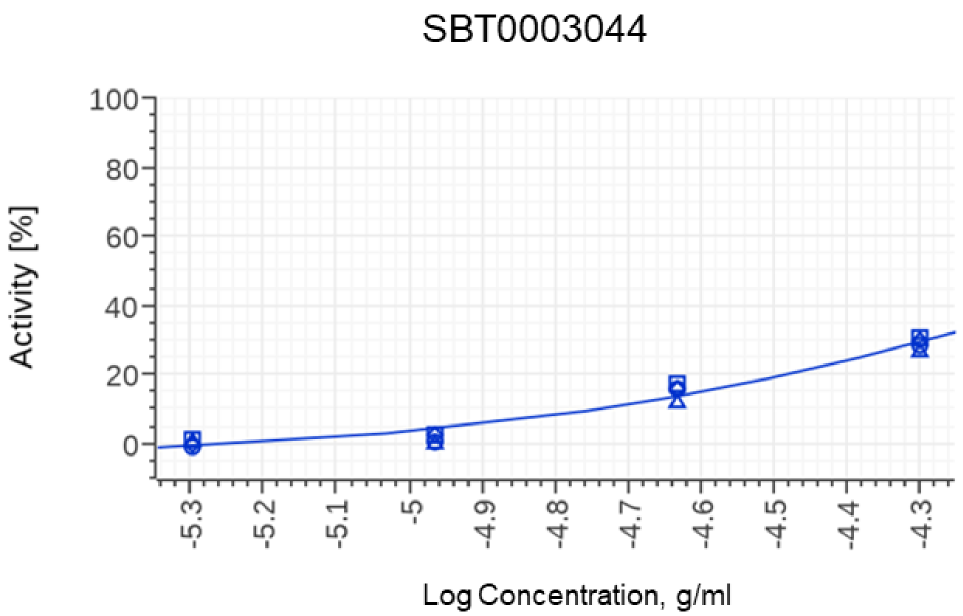

SI Figure 3 - Example of an extract tested in primary screening in quadruplicate at increasing concentrations and retrieved as active on PPARα assay (cut-off: mean plus 3 standard deviations of the distribution of % activity of Vehicle Control wells).
